# Nonlinear and symptom specific associations between chronotype and depression

**DOI:** 10.1038/s41598-024-79868-0

**Published:** 2024-11-20

**Authors:** Lennart Seizer, Estefanía Martínez-Albert, Johanna Löchner

**Affiliations:** 1 Department of Child and Adolescent Psychiatry, Psychosomatics and Psychotherapy, University Hospital of Tübingen, Tübingen, Germany; 2 German Center for Mental Health (DZPG), Tübingen, Germany; 3 Institute of Medical Psychology and Behavioural Neurobiology, University Hospital of Tübingen, Tübingen, Germany

**Keywords:** Chronotype, Sleep, Inflammation, Depression, Symptom, Translational research, Outcomes research, Human behaviour

## Abstract

The chronotype of individuals has been found to be predictive of depression risk and associated with the severity of depression. However, since depression is a phenotypically heterogeneous disease, it seems improbable that chronotype plays a role in every instance of depression. This study investigates the association between the two, while considering possible symptom-specificity and non-linearity of the relationship, utilizing a large sample from the National Health and Nutrition Examination Survey (*N* = 5217; 54% female; Age: *M* = 52.65, *SD* = 18.76). Depression symptoms were assessed using the Patient Health Questionnaire-9 (PHQ-9), and chronotype was determined by calculating sleep midpoints. Further, we also explored the potential mediating role of systemic inflammation, measured by C-reactive protein (CRP) levels, in the chronotype-depression link. The findings substantiate previous research indicating late chronotypes to be associated with higher PHQ-9 sum scores, with a minimum in PHQ-9 at a sleep midpoint of 02:49. The study further differentiates between individual depression symptoms, uncovering varying patterns of association with chronotype. No significant effect of chronotype on levels of CRP was found, suggesting that the link between chronotype and depression symptoms may not be directly mediated and appears to be stable and independent from systemic inflammation. The study highlights the non-linear and symptom-specific nature of the chronotype-depression connection and suggests the need for further, longitudinal studies to elucidate causal mechanisms and potential mediators.

## Introduction

Chronotype describes the temporal pattern of individual human behaviors, particularly the propensity when to wake and be active, and when to sleep, within a 24-h period^1^. This circadian inclination is relatively stable within subjects over time^2^, but can differ between subjects and is often described as a later chronotype (eveningness; late bedtime—late wake), an earlier chronotype (morningness; early bedtime—early wake), or somewhere in between^3^. Chronotypes are approximately normally distributed in the population with 25% having a sleep midpoint (i. e., the time halfway between bedtime and wakeup) earlier than 2:24, 50% between 2:24 and 4:15, and another 25% later than 4:15^4^. The chronotype of individuals has been cross-sectionally associated with having a depression disorder diagnosis and the severity of depression symptoms. Specifically, those with a later chronotype show more severe depression symptoms^5–7^. This association between depressive symptoms and delayed chronotype, has been observed in individuals with bipolar disorder, major depressive disorder, and even in community samples^8–10^. Further, longitudinal studies have found that the chronotype may be predictive of depression risk over periods of up to four years^11,12^ and that changes in chronotype over time correspond to changes in depressive symptoms^2^.

The link between chronotype and depression, as well as the directionality of effects, is not yet fully understood, but several potential mechanisms have been discussed. For example, rumination is associated with depression and individuals tend to engage in rumination more frequently towards the end of the day^13,14^. When one’s day is prolonged due to a late chronotype and delay in bedtime, there is an increased opportunity for rumination, potentially exacerbating symptoms of depression^6,15^. Further, depression is often accompanied with social withdrawal that includes patients leaving their homes less often and thus exposing themselves to less daylight^16^. As the chronotype is influenced by light exposure and low light conditions can lead to later chronotypes, depression and social withdrawal may subtly change the individual chronotype^17^. The potential mechanisms also include genetics and neuroendocrine systems: Alterations in clock genes and impaired hypothalamic–pituitary–adrenal or melatonergic functioning have been associated with distortion in sleep and circadian rhythm, as well as decreased mental health^18–21^. Moreover, systemic inflammation has been associated with both, later chronotype^22–24^ and depression symptoms^25–27^. Thus, systemic inflammation may represent a biological mechanism that mediates the association between chronotype and depression symptoms.

This study investigates the associations between chronotype, depression, and systemic inflammation. Previous research has mostly used composite scales of standardized depression questionnaires that aggregate various symptoms into a single score. This could introduce bias for two reasons. First, many depression screening tools include sleep disturbances, sleep quality, fatigue, and the ability to concentrate as symptoms of depression. These areas may be impaired due to depression, but are also more likely to be conspicuous in late chronotypes with increased sleep deprivation regardless of the presence of depression. It is conceivable that individuals with late chronotypes score higher in these questionnaires regardless of any other depression symptoms, such as anhedonia or loss of interest^28,29^. Second, depression is a phenotypically heterogeneous disease and it seems improbable that chronotype plays a role in every instance of depression, but may rather be connected to a subset of possible symptoms. A symptom-level approach could offer insights into the depression phenotypes associated with certain chronotypes, the mechanisms underlying depression linked to chronotype, and help guide patient selection in targeted sleep intervention and chronotherapy trials. Moreover, recent studies indicate the relation between chronotype and depression may be non-linear in nature with depressive symptoms increasing only after a certain threshold in sleep midpoints^30,31^, or follow a quadratic relation with increased depression symptoms toward earlier and later midpoints^32^. Thus, building on the existing research that established a link between chronotype and depression, in the current study, we will investigate the relation between chronotype and depression on a symptom-level while considering non-linear associations. Further, systemic inflammation will be tested as a potential mediator, linking the effects of chronotype on depression symptoms.

## Methods

### Study sample

The National Health and Nutrition Examination Survey (NHANES) samples are comprehensive, nationally representative surveys of the U.S. population, conducted by the National Center for Health Statistics (NCHS), a division of the Centers for Disease Control and Prevention (CDC). These surveys aim to assess a broad spectrum of physical and mental health indicators. The NCHS is responsible for the entirety of the data collection process and has given its official approval for the NHANES study protocols. Since 1999, NHANES has been performing continuous 2-year cycles of surveys involving a representative sample of the U.S. population. In each cycle, a new group of participants is selected. Individuals who were chosen and agreed to participate first underwent a computer-assisted interview conducted by trained staff in their homes. After this initial interview, all tests, including blood draws, as well as further interviews, such as those assessing depressive symptoms, were conducted at mobile examination centers. Detailed information on the survey design and methods can be found at https://wwwn.cdc.gov/nchs/nhanes. In this analysis, the 2017–2020 (pre-COVID 19 pandemic data) sample was used as it includes a questionnaire on sleep and chronotype and a screening tool on depression symptoms. Participants with missing data in these two questionnaires were removed from the sample (*n* = 571). Further, all participants who reported their working schedule included working evenings, nights, or early mornings during the past three months were excluded. Therefore, the final sample size utilized for the analysis spanned 5217 adults (54% female). The average age of the participants was 52.65 years (*SD* = 18.76), ranging from 18 to 80. Regarding the samples’ education, 25% of the participants reported their highest level of education to be a high school diploma, while 31% hold an associate or undergraduate college degree, 26% hold a college graduate degree or above, and 19% did not finish high school. 58% of the sample reported to be married and/or currently living with a partner. The participants reported an average usual sleep duration of 7.77 (*SD* = 1.66) hours per night on weekdays or workdays, and an average usual sleep duration of 8.24 (*SD* = 1.78) hours per night on weekends or non-workdays. The average nightly sleep duration per week ranged from 2 to 14 h. 31% reported they have previously talked to a doctor or other health professional about having trouble sleeping. For a more detailed description of the sleep duration in the NHANES samples, see^33^.

### Depression symptoms

The Patient Health Questionnaire-9 (PHQ-9) is a self-report tool consisting of nine items that was used to assess how frequently diagnostic criteria from the Diagnostic and Statistical Manual of Mental Disorders (DSM-IV) were met in the previous two weeks^34,35^. The nine items include the symptoms anhedonia, depressive mood, sleeping problems, fatigue, appetite changes, feeling inadequate, cognitive problems, psychomotor changes, and suicidal ideation. Each item was rated by the participants on a 4-point Likert scale, with 0 representing “Not at all”, 1 representing “Several days”, 2 representing “More than half the days”, and 3 representing “Nearly every day”. Regarding severity, the PHQ-9 sum score differentiates five categories, where scores of 0–4 indicate no depressive symptoms, 5–9 mild depressive symptoms, 10–14 moderate depressive symptoms, 15–19 moderately severe depressive symptoms, and 20–27 severe depressive symptoms^35^. The PHQ-9 has a good internal reliability with a Cronbach’s $\alpha$ of 0.89 and a good test–retest reliability with a Pearson correlation of 0.84 within 48 h^35^.

### Chronotype

The chronotype of participants was assessed using a short form of the Munich Chronotype Questionnaire^36^, a tool designed to evaluate an individual’s temporal preference for activity and sleep phases within a 24-h period. The questionnaire collected information about the sleep habits on workdays and free days, including sleep onset, sleep duration, and wake times. It showed good test–retest reliability within different time frames (14 days: Pearson’s r = 0.79; 60 days: Pearson’s r = 0.77)^36^. Chronotype was quantified based on the midpoint of sleep corrected for sleep debt accumulated during workdays. If for an individual the sleep duration on workdays was longer or equal to the sleep duration on free days, the midpoint of sleep on free days was used. If the sleep duration on workdays was shorter than the sleep duration on free days, the midpoint of sleep on free days was adjusted by subtracting half the difference between the sleep duration on free days and the weekly average in sleep duration^36^. This procedure accounts for sleep-debt accumulated over the workweek and sleep recovery on free days, which leads to variations in sleep timing between workdays and free days. We assumed five workdays and two free days per week for all participants^32,37^. The use of an alarm clock was not considered.

Note that we have used a mid-sleep measure rather than a measure that assesses the personal preference of morningness–eveningness, because mid-sleep provides a more accurate reflection of actual sleep behavior rather than just preferences. Preference measures may not align with actual behavior, leading to potential inaccuracies when studying the relationship between chronotype and depression^6^.

### Systemic inflammation

Blood samples were collected via venipuncture and were frozen at $-70 ^{\circ }$C until the day of the assay. Samples were analyzed for C-reactive protein (CRP) with a Roche Cobas 6000 chemistry analyzer, as a measure of systemic inflammation. The lower limit of detection (LLOD) was 0.15 mg/L, with levels below this value set to 0.11 mg/L, as determined by the formula LLOD/$\sqrt{2}$.

### Covariates

Several sociodemographic, lifestyle, and health-related covariates were considered in the estimated models. Basic sociodemographics were included as age in years, sex as a binary variable indicating male or female, and the socioeconomic status as the ratio of reported monthly income to the poverty index, specific to the family size, the appropriate year and the state of residence, following the U.S. Department of Health and Human Services’ guidelines. Health-related variables included the body mass index (BMI) and potential sleeping disorders as indicated by seeking professional help. Lifestyle factors included the quantity of alcohol consumption during the past year as an eleven-point Likert-scale ranging from 1 = “Every day” to 11 = “Never”, and the average sleep duration (in hours) per week. Further details on covariate measurements are available at https://wwwn.cdc.gov/nchs/nhanes and descriptive statistics for the current sample are given in Table 1.

**Table 1:** Sample descriptives and Pearson correlation matrix

	Mean	SD	N	1	2	3	4	5	6	7
1. Age	52.65	18.76	5216							
2. BMI	29.99	7.67	5136	0.04^b^						
3. SES	2.35	1.58	4175	0.08^b^	− 0.07^b^					
4. Alcohol intake	7.49	3.08	4628	0.11^b^	0.10^b^	− 0.16^b^				
5. Sleep midpoint	03:16	01:34	5185	− 0.32^b^	0.00	− 0.07^b^	− 0.02^a^			
6. Sleep duration	7.90	1.57	5216	− 0.11^b^	− 0.15^b^	0.01	− 0.04^b^	− 0.03^a^		
7. PHQ-9	3.51	4.46	5216	− 0.03^a^	0.12^b^	− 0.18^b^	0.05^b^	0.14^b^	− 0.36^b^	
8. CRP	4.35	9.37	4835	0.04^a^	0.24^b^	− 0.08^b^	0.07^b^	0.02	− 0.07^b^	0.09^b^

The measurement of the variables is described in the Methods section. BMI, Body mass index; SES, Socioeconomic status; CRP, C-reactive protein; PHQ-9, Patient health questionnaire-9; SD, Standard deviation. ^a^*p* < 0.05. ^b^*p* < 0.01.

### Data analysis

All statistical analyses were performed using *R* 4.4^38^. To estimate the association of chronotype with individual depression symptoms and sum scores, ordered logistic regression models and linear regression models were used respectively. The models included the following covariates: age, sex, socioeconomic status, average sleep duration, alcohol use, and potential sleep disorders. To investigate potential non-linear effects, natural cubic splines were used in the models. Splines enable the modeling of non-linear trajectories using knots to split the trajectory into segments. To alter the amount of knots, we changed the degrees of freedom from 1 to 10 for each PHQ symptom model and extracted the Akaike Information Criterion (AIC) to derive the ideal number of degrees of freedom. The goal of information-based model selection is to find the most accurate models while making sure they are as parsimonious as possible. We used the corresponding AIC values to estimate model weights in order to determine which of the candidate spline models provided the best fit^39,40^. Model diagnostics included checking the distribution and homogeneity of the residuals, the presence of influential observations, and the collinearity of predictors. For the ordinal logistic regressions, diagnostics were performed using surrogate residuals^41^. Further, the effect of chronotype on CRP was estimated using generalized regression models with Gamma log-link distributions, and BMI as an additional covariate to the described. Prior to the analyses, outliers in chronotype, defined as data points that are outside three interquartile ranges from the central 50% of the data, were removed (*n* = 20).

## Results

**Fig. 1:**
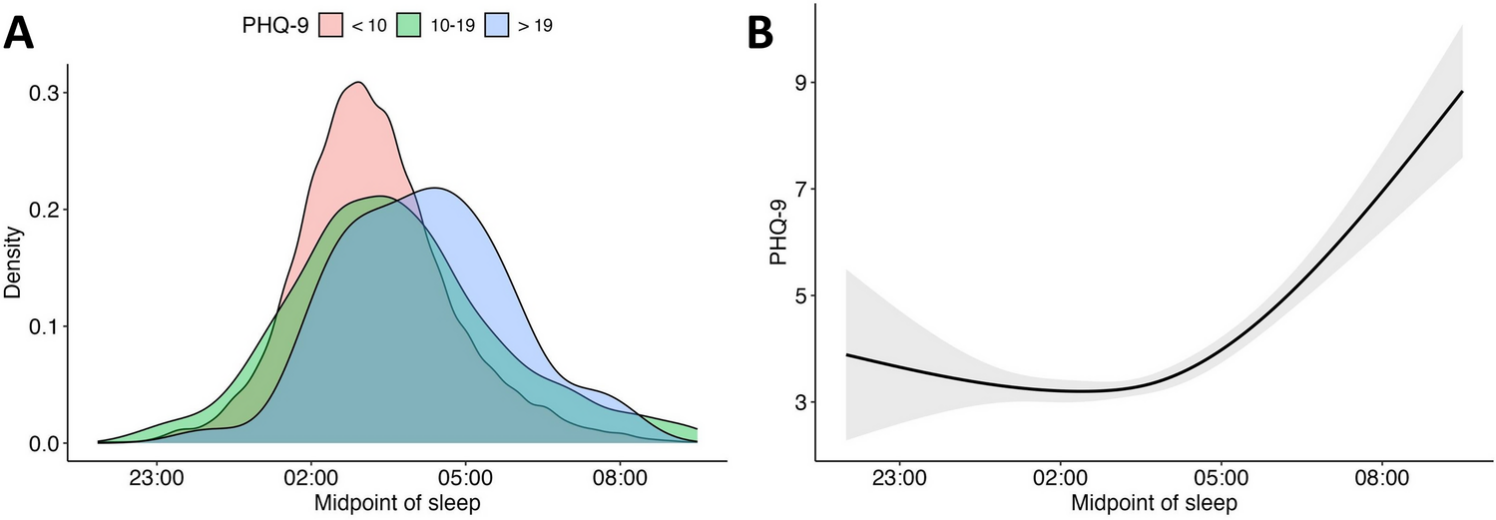
Association between PHQ-9 sum scores and chronotype.

The distribution of sleep midpoints across different PHQ-9 sum scores is illustrated in Fig. 1A. Specifically, participants have been grouped into groups with mild PHQ-9 scores (< 10; *N* = 4655), moderate to moderately severe PHQ-9 scores (10–19; *N* = 507), and severe PHQ-9 scores (>19; *N* = 55). The group of participants with low PHQ-9 scores showed significantly earlier sleep midpoints (*M* = 03:13, *SD* = 01:30) compared to participants with medium PHQ-9 scores (*M* = 03:38, *SD* = 02:02; *t* = 4.35, *p* < .001, *d* = 0.19) and participants with high PHQ-9 scores (*M* = 04:12, *SD* = 01:39; *t* = 4.40, *p* < .001, *d* = 0.59). Further, individuals with medium PHQ-9 scores showed significantly earlier sleep midpoints compared to individuals with high PHQ-9 scores (*t* = 2.40, *p* < .019, *d* = 0.28). Moreover, we found a non-linear effect of sleep midpoints on PHQ sum scores in a natural cubic spline regression with three degrees of freedom (*Adj. R*^2^ = 0.19, *F* = 68.24, *p* < .001). PHQ-9 scores reached their lowest point at a sleep midpoint of 02:49 with a weak increase towards earlier midpoints and a strong increase towards later midpoints (Fig. 1B).

**Fig. 2:**
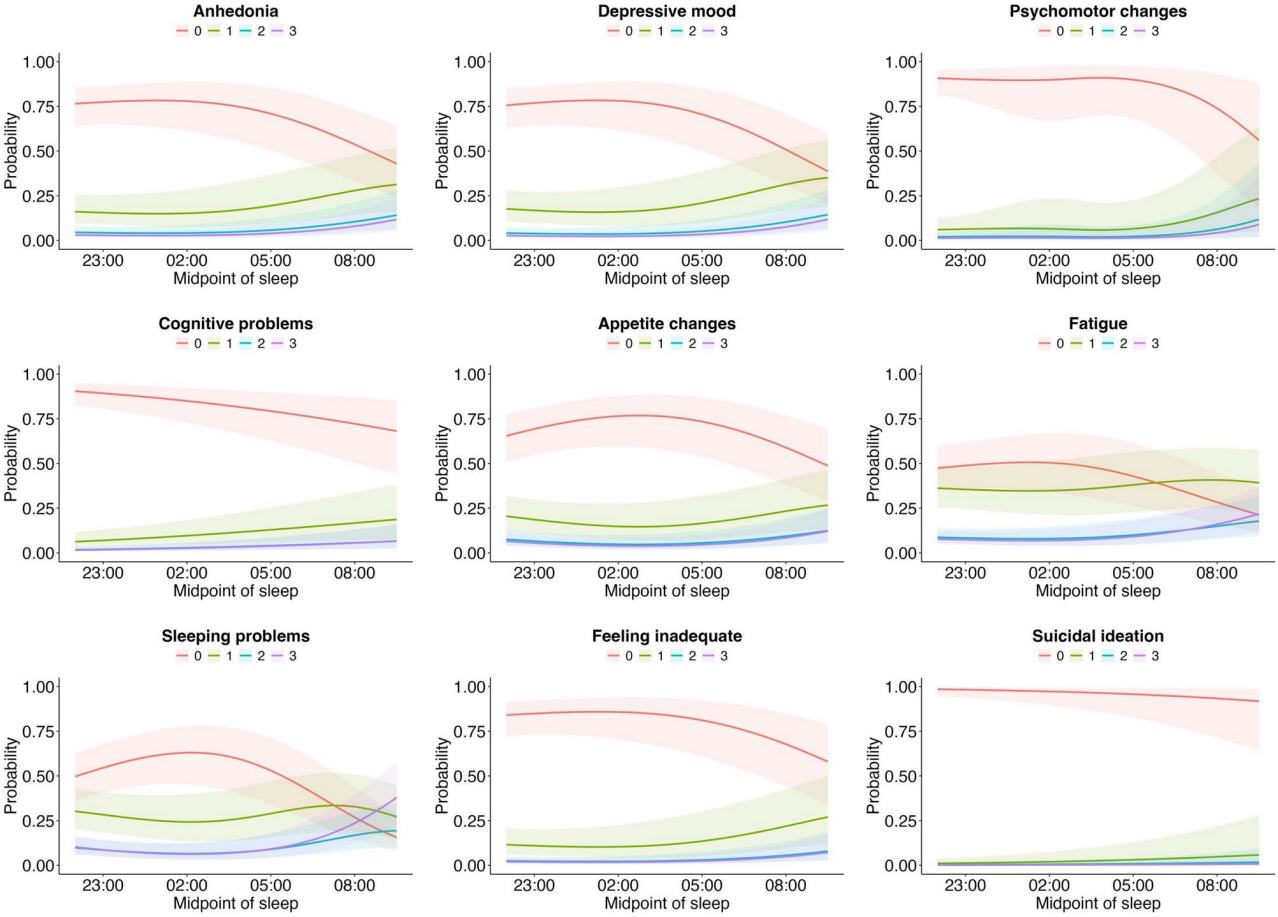
Symptom-level association between PHQ-9 and chronotype. The plots show the probability for the Likert-scaled PHQ-9 item answers as a function of sleep midpoint with 0 representing “Not at all”, 1 representing “Several days”, 2 representing “More than half the days”, and 3 representing “Nearly every day”.

Figure 2 shows the associations of chronotype with individual depressive symptoms (i.e., the PHQ-9 items) as a result of the ordered logistic regressions. The best fit for each model was achieved by including natural cubic splines with one to three degrees of freedom, as determined by AIC. For each symptom, the probability of the Likert-scale answers is given as a function of chronotype. For example, in the upper left plot for anhedonia, we see that the probability of answering “Not at all” decreases for chronotypes with a sleep midpoint later than 02:00, while the probabilities for answering “Several days”, “More than half the days”, and “Nearly every day” increases.

In the generalized regression model, there was no significant effect of chronotype on CRP (*b* = 0.01, *p* = 0.78). In sensitivity analyses, CRP values were winsorized to 5, 10, and 15 mg/L, but none of these adjustments changed the significance of the results.

## Discussion

The study found significant associations between a later midpoint of sleep and various symptoms of depression, even after adjusting for sociodemographic, lifestyle, and health-related factors. The results suggest a non-linear relationship between sleep midpoints and PHQ-9 sum scores, with a minimum in PHQ-9 scores around a sleep midpoint of 02:49, indicating that both early and late midpoints of sleep are associated with higher depression scores, although the increase is more pronounced for later midpoints. This fits to previous studies that found a positive correlation between chronotype and depression symptoms only for sleep midpoints later than 01:00^31^ or 02:18^30^; and to studies that found increased depression symptoms in individuals with sleep midpoints outside a range of 02:00 and 04:00^42^ and an increased prevalence and risk of depression in both early and late chronotypes^32^.

Further, a similar relationship was found between chronotype and the individual depression symptoms, although there were considerable differences in the effect pattern of individual symptoms. For example, while the probability to experience cognitive problems increased linearly with later chronotype, the probability to have psychomotor changes remained stable until a sleep midpoint of about 06:00 when it declined sharply. Previous research indicates that chronotype is strongly related to cognitive functioning, with better performance at the individual’s preferred time of the day^43^, and psychomotor performance decrement has been shown in evening-type but not in morning-type individuals^44^. In our results, the probability for appetite changes increased toward both earlier and later chronotypes. This has been observed especially for late chronotypes since those individuals often report to have greater evening food intake or binge eating^45,46^. Furthermore, a recent study showed that both, morningness and eveningness, are related to emotional eating, depression, anxiety, stress, and life satisfaction^47^. Regarding the probability of suicidal ideation, this remained relatively unaffected by variation in chronotype, which is consistent with earlier studies where individuals with different chronotypes did not differ in their self-reported experiences of suicidal ideation or past suicide attempts^48^.

The relationship of chronotype with sleeping problems and fatigue is more complex as chronotype is closely linked to sleep-wake patterns, sleep duration, and poor sleep quality^49,50^. Thus, the analysis was adjusted by sleep duration and potential sleep disorders. We show that the probability of experiencing both, sleep problems and fatigue, is higher in late chronotypes. Sleep disturbances have been proposed as mediators between eveningness and depression since these disturbances may cause depression or be caused by depression^51^. In this context, there have been contrary findings regarding the role of sleep quality in the association between chronotype and depression^9,52^, with some finding sleep quality mediating this relationship, while others find that evening preference is associated with depressive states independently of sleep duration, sleep quality, sleep timing, and sleep debt. In view of our results these contradictions may be explained by the fact that different depression symptoms show various relations to chronotype, and thus sleep quality may be involved to varying degrees with each of these.

The underlying mechanisms linking chronotype and depression are still unknown. One suggested mechanism is an increase in inflammatory markers. There is evidence suggesting that chronotype may influence inflammation levels in the body. Previous studies have found that evening chronotypes have higher levels of inflammatory markers compared to morning chronotypes^22,53^. Further, depression has been also associated with increased inflammation in the body, as elevated levels of inflammatory markers have been found in individuals with depression^54^. In the current study, no significant effect of chronotype on levels of CRP was found in our study, suggesting that the link between chronotype and depression symptoms may not be directly mediated and appears to be stable and independent from systemic inflammation.

The study possesses several strengths, including the use of a large sample, nationally representative of the US adult population, which enhances the generalizability of the findings. The assessment of chronotype as sleep-dept corrected MSF, coupled with the consideration of a wide range of covariates, adds to the robustness of the analysis. However, there are limitations to note. First, the cross-sectional design limits the ability to infer causality between chronotype and depression symptoms, e.g., as it remains unclear, whether aberrant sleep midpoints during depressive episodes would evolve back in remission. Longitudinal studies will be necessary to investigate the effect of chronotype as depression symptoms increase or decrease, for example before, during, and after a depressive episode^55,56^. Additionally, many symptoms of depression are complex and not adequately evaluated by brief measures such as the PHQ-9. For example, some PHQ-9 items cover various extremes of symptoms, such as changes in appetite (increases and decreases) and psychomotor changes (agitation and retardation), and it remains uncertain from this study whether the predictors are linked to only one extreme of these symptoms. A more comprehensive depression assessment tool could potentially capture further subtleties in the depression phenotype. Further, while CRP is a commonly employed marker of inflammation in depression research, regarding the multitude of proteins implicated in inflammation, it is imperative to consider a variety of other inflammatory proteins to allow for meaningful results on the relation between inflammation, depression, and chronotype. Finally, the exclusion of individuals with atypical work schedules may have omitted a population with potentially different sleep and depression dynamics, possibly affecting the generalizability of the findings.

In conclusion, this study contributes to the growing body of evidence on the association between sleep patterns, specifically chronotype, and depression. The findings underscore the importance of considering individual differences in sleep timing in the context of mental health. The absence of a link between chronotype and systemic inflammation as measured by CRP levels indicates that other mechanisms may underlie the relationship between sleep patterns and depression symptoms. Further research, particularly longitudinal studies^57^, is needed to explore these dynamics more deeply, including the potential causal pathways and the role of lifestyle and environmental factors in mediating these relationships.

## Data Availability

The data are available at https://wwwn.cdc.gov/nchs/nhanes.
